# The Warburg Effect Redefined: A Kinetic and Regulatory Perspective

**DOI:** 10.7759/cureus.93331

**Published:** 2025-09-27

**Authors:** Amal Bhanu Vayakkattil, Aiswarya S Pazhanchery, Shibu Andrews, Udayabhanu Vayakkattil

**Affiliations:** 1 Biochemistry, Sri Devaraj Urs Medical College, Kolar, IND; 2 Pediatric Medicine, Kottayam Medical College, Kottayam, IND; 3 Orthopaedics, Government Medical College Thrissur, Thrissur, IND; 4 Biochemistry, Gamma High Tech Laboratory, Thrissur, IND

**Keywords:** atp, cancer, cell proliferation, epigenetic, glucose transporter, glycolysis, lactate, lactate dehydrogenase, monocarboxylate transporters, warburg effect

## Abstract

The Warburg effect, characterized by the preferential conversion of glucose to lactate despite adequate oxygen availability, constitutes a regulated metabolic adaptation rather than a mere dysfunctional response to hypoxia. This metabolic shift arises because lactate dehydrogenase (LDH) exhibits a significantly higher catalytic capacity compared to pyruvate dehydrogenase (PDH), resulting in a substantial reduction of pyruvate to lactate once PDH becomes saturated. In cancer cells, this kinetic preference is further amplified by the upregulation of glucose and monocarboxylate transporters (GLUT1, MCT1, and MCT4) and alterations to the plasma membrane, which enhance transport efficiency. These adaptations maintain a high glycolytic flux, facilitate continuous lactate efflux, and circumvent traditional feedback inhibition. The accumulation of glycolytic intermediates supports the biosynthesis of nucleotides, lipids, and proteins, thereby promoting tumorigenesis. Over time, metabolite-induced DNA methylation and chromatin remodeling reinforce this metabolic state, stabilizing the oxygen-independent proliferative phenotype. Consequently, the Warburg effect is best conceptualized as a primary metabolic strategy initiated by membrane remodeling, sustained by kinetic flux imbalances, and perpetuated by epigenetic feedback, collectively enabling tumor growth and survival in adverse microenvironments.

## Introduction and background

The Warburg effect has long posed a significant question in cancer biology [1]: Why do rapidly proliferating cancer cells preferentially engage in glycolysis, a less efficient pathway for adenosine triphosphate (ATP) production per glucose molecule, even when sufficient oxygen is available to support oxidative phosphorylation (OXPHOS)? Although mitochondrial respiration yields significantly more ATP per glucose molecule, glycolysis proceeds at a much faster rate, facilitating rapid ATP generation [1,2]. This phenomenon is analogous to the physiological adaptation observed in skeletal muscles during intense exercise, where anaerobic glycolysis temporarily predominates to meet the acute energy demands. However, the persistent reliance of cancer cells on glycolysis under normoxic conditions suggests a more complex and regulated metabolic framework, beyond mere energy economics. Traditional explanations, such as mitochondrial dysfunction, oncogene-driven metabolic reprogramming, and isolated genetic mutations, do not adequately account for the coordinated, stable, and self-sustaining nature of cancer cell metabolism. We propose a shift from a reductionist approach to a systems-level perspective, wherein the Warburg effect is perceived not as a metabolic defect but as a robust and adaptive strategy optimized to meet the complex demands of proliferating cells, including malignant cells. This strategy involves highly coordinated changes across multiple levels. Overexpression of glucose transporter 1 (GLUT1) ensures sustained glucose uptake while bypassing normal feedback inhibition. Concurrent upregulation of monocarboxylate transporters MCT1 and MCT4 [3] facilitates continuous lactate export, thereby preventing intracellular lactate accumulation and metabolic feedback inhibition by lactate. Structural remodeling of the plasma membrane, including increased fluidity and altered electrostatic properties, enhances transporter function and nutrient flux. The high glycolytic flux reveals a key kinetic property of the pathway; its enzymes do not uniformly match the catalytic capacity. Under normal conditions, this imbalance poses a minimal risk. However, in cancer cells, accelerated flux leads to the accumulation of specific intermediates at the enzymatic bottlenecks. When these accumulated intermediates serve as substrates for anabolic branching points, they divert to biosynthetic pathways that support the production of nucleotides, amino acids, lipids, and other macromolecules required for rapid cell proliferation [4]. Thus, accumulation of intermediates at key metabolic branching points triggers anabolic reprogramming. This enables cancer cells to generate ATP while simultaneously producing the molecular building blocks required for biomass expansion, thereby supporting sustained growth and division. Over time, this metabolic phenotype has been stabilized by epigenetic modifications [5], including DNA methylation, histone modifications, and non-coding RNA activity. These changes ensure that daughter cells inherit a pro-glycolytic biosynthetic state. What begins as a flexible adaptation to environmental cues ultimately evolves into a locked-in, self-perpetuating system that sustains malignancy.

Therefore, the Warburg effect is best understood as an upstream metabolic adaptation, rather than a downstream consequence of genetic mutations. It originates from changes in membrane composition and transporter expression, is driven by the kinetic advantage of lactate dehydrogenase (LDH) over pyruvate dehydrogenase (PDH), and is stabilized by epigenetic mechanisms. This integrated regulatory network enables cancer cells to maintain a continuous ATP production by upregulating glucose uptake and lactate export. These adaptations allow cancer cells to thrive in hostile microenvironments and sustain relentless growth and survival.

## Review

Review methodology

This narrative, concept-driven review aims to establish an integrated kinetic and regulatory framework for the Warburg effect. As the objective was theoretical synthesis rather than addressing a narrowly defined clinical question, formal systematic protocols such as Preferred Reporting Items for Systematic Reviews and Meta-Analyses (PRISMA) were not employed. Instead, methodological rigor was maintained in accordance with the standards for high-quality narrative reviews. Relevant literature was identified through searches of PubMed, Scopus, and Web of Science from January 2000 to August 2025. The search terms encompassed the core concept (“Warburg effect” OR “aerobic glycolysis”), enzyme kinetics (LDH, PDH, Vmax, Km), transport mechanisms (GLUT1/SLC2A1, MCT1/SLC16A1, MCT4/SLC16A3, lactate transport), regulatory pathways (PFK1, PFKFB3, ATP, citrate, AMPK, mTOR, HIF-1α, MYC), anabolic branching (pentose phosphate pathway, serine/glycine, lipid and nucleotide synthesis), and stabilization mechanisms (epigenetics, chromatin remodeling, histone lactylation). The inclusion criteria prioritized peer-reviewed original studies and seminal reviews that reported enzyme kinetics, metabolic fluxes, transporter function, or connections between glycolysis and anabolic or epigenetic regulation. Grey literature, non-peer-reviewed sources, and studies lacking mechanistic insights were excluded. Screening included backward and forward citation tracking to ensure comprehensive coverage and to minimize bias. The findings were synthesized into a four-stage framework - kinetic, transport, anabolic, and epigenetic - which constitutes the basis of the proposed redefinition of the Warburg effect.

Regulation and Dynamics of Glycolytic Flux in Healthy Cells

In healthy cells, glycolysis is a tightly regulated process that aligns glucose utilization with the energy and biosynthetic demands of the cell. Glucose, a polar molecule, cannot cross the hydrophobic lipid bilayer of the plasma membrane freely. Even when a favorable concentration gradient exists, it cannot diffuse passively into the cells. To overcome this barrier, cells use specific transporter proteins such as GLUT1, which facilitate glucose entry into the cytoplasm in a controlled manner.

Upon entering the cell, glucose undergoes a 10-step enzymatic process, termed glycolysis, culminating in the formation of two pyruvate molecules and a net gain of two ATP molecules per glucose molecule. Most cells possess a robust and efficient glycolytic enzyme system capable of processing the incoming glucose loads. The fate of pyruvate is determined by the relative activity of two competing enzymatic pathways: oxidative decarboxylation to acetyl-CoA via the PDH complex or reduction to lactate via LDH. Although PDH exhibits a higher affinity for pyruvate and reacts more rapidly, the overall flux is influenced not only by substrate affinity but also by kinetic capacity (Vmax) and the rate of product clearance.

At low intracellular pyruvate concentrations, PDH efficiently channels pyruvate into the mitochondria for oxidative metabolism. However, during periods of elevated glycolytic flux, pyruvate production surpasses the processing capacity of PDH, leading to its intracellular accumulation. Under these conditions, LDH becomes increasingly active and converts excess pyruvate into lactate. This reaction prevents pyruvate accumulation and regenerates NAD⁺, an essential cofactor for NAD⁺-dependent glycolytic enzymes such as glyceraldehyde-3-phosphate dehydrogenase (GAPDH). Although the mitochondrial respiratory chain can regenerate NAD⁺ via OXPHOS, its capacity is insufficient to sustain the exceptionally high glycolytic flux conditions [1].

LDH, characterized by a notably higher catalytic turnover rate than PDH, facilitates the efficient clearance of excess pyruvate. The continuous export of lactate via the monocarboxylate transporters MCT1 and MCT4 is essential for maintaining low intracellular lactate concentrations, which are critical for preventing product inhibition and ensuring the forward progression of the LDH-catalyzed reaction [6]. Empirical evidence indicates that lactate can directly inhibit phosphofructokinase 1 (PFK1) activity by promoting tyrosine phosphorylation in skeletal muscles, even in the presence of insulin. Furthermore, lactate indirectly suppresses hexokinase (HK) activity by inducing conformational changes that impair glucose phosphorylation and reduce glycolytic commitment [7]. Thus, efficient lactate clearance is essential for sustaining NAD⁺ regeneration and preserving the functional integrity of the key glycolytic enzymes. By continuously exporting lactate, the accumulation and inhibition of products are reduced, thereby maintaining a steady glycolytic flux, particularly when metabolic demand is high.

Although OXPHOS yields significantly more ATP per glucose molecule, the combination of glycolysis and LDH activity enables faster ATP production via substrate-level phosphorylation. This fermentative pathway has a higher enzymatic turnover capacity, and in some contexts, individual glycolytic enzymes operate at rates several-fold to over 100 times faster than mitochondrial complexes [8]. When evaluated by ATP production per unit time, glycolysis can generate ATP several times faster than OXPHOS, making it advantageous during periods of acute or fluctuating energy demand.

Importantly, the high glycolytic throughput is not limited to hypoxia. It also occurs in well-oxygenated tissues with fluctuating or elevated energy demands and is regulated by the Vmax values of PDH and LDH. For example, during strenuous exercise, skeletal muscles produce lactate not only when oxygen delivery is insufficient but also when oxygen is available. In such cases, the glycolysis rate exceeds the mitochondrial oxidative capacity, resulting in the partial diversion of pyruvate to lactate [9]. Similarly, many proliferating cells, including normal and cancerous cells, convert a portion of pyruvate to lactate despite having intact mitochondrial respiration [10].

Feedback Mechanisms and Cellular Homeostasis

Glycolysis is not a strictly linear sequence but a flexible metabolic network with multiple branching points. These branches allow carbon intermediates to divert into various biosynthetic pathways, enabling cells to adapt to the shifting energy and anabolic requirements. Under normal physiological conditions, core glycolytic enzymes exhibit a higher catalytic efficiency than those that initiate branching pathways. Consequently, most branches remain relatively inactive unless specific metabolic changes occur, such as the build-up of intermediates due to glycolytic bottlenecks or sustained increases in glycolytic flux [4].

A fundamental regulatory characteristic of normal cells is the stringent control of glycolytic flux through feedback mechanisms that synchronize nutrient consumption with the cell’s energy and redox status, extending beyond product inhibition. PFK1, which catalyzes the conversion of fructose-6-phosphate (F6P) to fructose-1,6-bisphosphate, functions as the central regulatory checkpoint. Its activity is allosterically inhibited by ATP, citrate, and lactate, reflecting conditions of energy sufficiency and a diminished requirement for further glycolytic throughput [11]. These molecules help restrain excessive glucose metabolism by reducing PFK1 activity.

ATP serves a dual regulatory role in glycolysis, functioning as both a substrate and an allosteric inhibitor of phosphofructokinase-1 (PFK1), a pivotal rate-limiting enzyme in this metabolic pathway. It engages with two distinct sites on PFK1: a catalytic site that facilitates the phosphorylation of F6P and a separate allosteric site that mediates feedback inhibition. Under conditions of high energy demand, ATP predominantly occupies catalytic sites, thereby promoting glycolytic flux. Conversely, when intracellular ATP concentrations exceed immediate metabolic requirements, ATP increasingly binds to the allosteric inhibitory site, thereby diminishing PFK1 activity and downregulating glycolysis [12]. This feedback mechanism enables cells to dynamically adjust their glycolytic throughput in response to energy availability, maintain metabolic homeostasis, and prevent ATP overproduction. Furthermore, ATP functions as a broad metabolic regulator by inhibiting multiple energy-generating pathways, including fatty acid β-oxidation, pyruvate kinase M2 (PKM2), and the PDH complex. These inhibitory effects further enhance the cell’s capacity to downregulate energy production when the ATP supply exceeds the demand, thereby sustaining metabolic homeostasis [12].

Citrate, an intermediate of the tricarboxylic acid (TCA) cycle, reinforces the feedback inhibition of glycolysis. When the mitochondrial oxidative metabolism becomes saturated, excess citrate is exported to the cytosol, enhancing ATP-mediated inhibition by allosterically stabilizing the inactive conformation of PFK1 [13]. In parallel, lactate accumulation during periods of elevated glycolytic flux contributes to feedback inhibition by downregulating the activity of both PFK1 and HK [6]. Together, these mechanisms exert a more comprehensive regulatory effect on glycolytic throughput than any individual feedback loop does.

Further downstream of glycolysis, PKM2, which catalyzes the conversion of phosphoenolpyruvate (PEP) to pyruvate, is also subject to allosteric feedback inhibition [2]. Elevated ATP levels suppress PKM2 activity, introducing a secondary regulatory point that limits pyruvate production and slows the glycolytic flux [14]. This results in the accumulation of upstream glycolytic intermediates, particularly when the ATP-mediated inhibition of PFK1 is inadequate. Accumulated intermediates such as 3PG are converted into serine and glycine, whereas DHAP contributes to the biosynthesis of glycerol and glycolipids.

Lactate, the terminal product of anaerobic glycolysis, also contributes to the redox-sensitive feedback loops. When the rate of pyruvate formation exceeds mitochondrial oxidative capacity, LDH reduces pyruvate to lactate, regenerates NAD^+^, and allows glycolysis to continue. However, if lactate export becomes insufficient and intracellular lactate accumulates, equilibrium shifts toward pyruvate. This reduces NAD⁺ regeneration, lowers the NAD^+^/ NADH ratio, inhibits GAPDH, and creates a bottleneck that slows glycolytic throughput [6,7].

Another feedback layer involves acetyl-CoA production via pyruvate and fatty acid β-oxidation. Under energy-rich conditions, elevated levels of acetyl-CoA, NADH, and ATP activate pyruvate dehydrogenase kinase (PDK), which phosphorylates and inhibits PDH [15]. High ATP and NADH levels suppress β-oxidation, prevent excessive acetyl-CoA accumulation, and limit the generation of reduced equivalents. Conversely, elevated β-oxidation increases mitochondrial acetyl-CoA and ATP levels, which further activate PDK, leading to PDH inhibition and reduced pyruvate oxidation.

These feedback mechanisms at multiple levels ensure that glycolytic flux, mitochondrial respiration, and lipid oxidation are coordinated with cellular energy demand. Simultaneously, the high-energy state supports anabolic processes by regulating the availability of metabolic intermediates and maintaining redox balance [13,15].

Metabolic Branching Into Anabolic Pathways

Metabolic branching often arises from a kinetic mismatch between enzymes. For example, HK, which converts glucose to glucose 6 phosphate (G6P), exhibits a high catalytic efficiency. Its product is then processed by phosphoglucose isomerase (PGI). However, under high glucose influx, PGI can become rate-limiting because of its low catalytic capacity. This mismatch leads to G6P accumulation. This effect was further amplified when PFK1 was inhibited by elevated ATP, citrate, or lactate levels, resulting in F6P accumulation. Because the PGI-catalyzed reaction is reversible, excess F6P drives G6P accumulation via a reverse reaction [11,12].

Although HK is subject to product inhibition by G6P, its high turnover rate delays the onset of suppression. This temporal lag allows for the transient accumulation of G6P, which acts as a substrate reservoir for the branching anabolic pathways. The pentose phosphate pathway (PPP) is typically initiated by glucose-6-phosphate dehydrogenase (G6PD). Owing to its higher affinity for G6P than for PGI, G6PD preferentially directs G6P toward PPP [16]. However, this pathway has limited capacity because of G6PD's relatively low catalytic efficiency and product inhibition by NADPH. Consequently, PPP operates more slowly than glycolysis under normal physiological conditions.

When F6P accumulates beyond the glycolytic capacity in the presence of excess ATP, it may be redirected into the hexosamine biosynthesis pathway, which supports glycoprotein and glycolipid synthesis [17]. Additionally, ATP inhibits pyruvate kinase M2 (PKM2), thereby diminishing the glycolytic output and fostering the accumulation of upstream intermediates within the glycolytic pathway. As metabolic flux intensifies, this sequential saturation of control points facilitates the activation of low-capacity anabolic biosynthetic pathways [2,14].

In cancer cells where glycolysis is persistently upregulated, both glycolytic intermediates and ATP accumulate at high levels. This enables the continuous support of anabolic processes. Glycolytic intermediates are redirected to nucleotide synthesis via PPP, amino acid biosynthesis via 3PG and pyruvate, and lipid synthesis via DHAP and acetyl-CoA.

This redistribution of carbon intermediates is not a passive overflow, but rather a tightly coordinated and regulated process. It integrates the cellular energy status, redox balance, and biosynthetic demands, ultimately supporting sustained growth and survival under proliferative conditions [2,4].

Pyruvate Partitioning and the Warburg Effect

The Warburg effect, which is characterized by preferential lactate production despite adequate oxygen availability, has long been regarded as a metabolic paradox in cancer biology. A clear rationale emerges when pyruvate partitioning in normal and metabolically active cells is examined in terms of energy homeostasis and redox balance.

Cells intrinsically regulate nutrient uptake and energy production to align their biosynthetic needs with their energetic demands. The metabolic fate of pyruvate is a critical determinant in the progression of glucose metabolism through either oxidative or fermentative pathways. This fate is primarily influenced by the relative activities of PDH and LDH. PDH irreversibly converts pyruvate to acetyl-CoA, thereby committing carbon to the TCA cycle and mitochondrial oxidative (OXPHOS). In contrast, LDH catalyzes the reversible reduction of pyruvate to lactate, simultaneously regenerating NAD^+^ to sustain glycolytic flux [15,18].

Under energy depletion conditions, the PDH complex actively directs pyruvate into the TCA cycle, resulting in the production of NADH and FADH₂. These molecules subsequently donate electrons to the mitochondrial respiratory chain, facilitating ATP synthesis [18]. PDH activity is dynamically regulated by phosphorylation by PDK and dephosphorylation by pyruvate dehydrogenase phosphatase (PDP) [19]. Elevated ATP, NADH, and acetyl-CoA levels activate PDK, thereby inhibiting PDH and promoting the conversion of pyruvate to lactate. Conversely, an increase in ADP and NAD⁺ stimulates PDP, thereby restoring PDH activity to support oxidative ATP synthesis [20]. This regulatory mechanism ensures that pyruvate utilization is primarily dictated by the intracellular energy status rather than oxygen tension.

In rapidly proliferating or highly active cells, glycolysis may surpass the oxidative capacity of the mitochondria, resulting in the accumulation of pyruvate within the cytoplasm. Although PDH exhibits high substrate affinity, its catalytic capacity is lower than that of LDH, which converts substantial amounts of excess pyruvate into lactate. This conversion prevents pyruvate accumulation and regenerates NAD⁺, a crucial cofactor for glyceraldehyde 3-phosphate dehydrogenase (GAPDH), thereby maintaining glycolytic flux. Although NAD^+^ can also be regenerated through OXPHOS, the mitochondrial capacity for NADH oxidation may be inadequate during periods of elevated glycolytic flux. Under such conditions, lactate production becomes essential for sustaining glycolysis, and the high catalytic efficiency of LDH is particularly well suited to fulfil this requirement [8,9].

Efficient lactate export is crucial for preventing intracellular acidification and sustaining forward progression of the LDH reaction. The accumulation of lactate and protons can induce product inhibition, shifting the reaction toward pyruvate, thereby diminishing the catalytic efficiency of LDH and hindering the regeneration of NAD^+^ during glycolysis. The monocarboxylate transporters MCT1 and MCT4 facilitate proton-coupled lactate efflux, thereby supporting cytosolic pH balance and the continuity of glycolysis. These transporters are often upregulated in cancer cells, enabling sustained and rapid clearance of lactate [3,21].

This regulated preference for lactate production in the presence of oxygen does not indicate mitochondrial dysfunction; rather, it represents an adaptive metabolic strategy. The increased uptake of glucose via GLUT1 and enhanced export of lactate through monocarboxylate transporters MCT1 and MCT4 facilitate rapid ATP generation while supplying glycolytic intermediates for biosynthetic pathways [21]. Although oxidative OXPHOS produces more ATP per glucose molecule, glycolysis can proceed at a rate up to 100 times faster [8], enabling proliferating cells to satisfy their immediate energy demands while supporting their anabolic growth. The consequent rise in ATP levels contributes to the suppression of PDH, ensuring that pyruvate is preferentially directed toward lactate production as long as the glycolytic flux remains elevated.

Potential Biophysical Triggers for the Warburg Effect

During the initial stages of oncogenic transformation, cells typically retain functional mitochondria and primarily rely on oxidative phosphorylation (OXPHOS) for ATP production before switching to the Warburg effect. The precise trigger for the metabolic shift from oxidative metabolism to aerobic glycolysis, known as the Warburg effect, remains unknown. Although oncogene activation (e.g., MYC and RAS) and hypoxia (HIF-1α stabilization) are acknowledged contributors, they do not fully explain the metabolic phenotype observed in all cancer types. We propose that metabolic reprogramming may also be initiated by alterations in the mechanical microenvironment of cells, potentially acting in conjunction with or even preceding traditional oncogenic signaling to induce a pro-glycolytic state in cancer cells. This perspective suggests that early biophysical changes in the tumor microenvironment may serve as critical upstream modulators. Relevant mechanical stimuli include increased interstitial hydrostatic pressure, enhanced extracellular matrix (ECM) stiffness, fluid shear stress, and altered tissue elasticity [22]. Under normal conditions, these forces regulate essential processes such as embryonic development and tissue repair through mechanotransduction. However, in pathological conditions such as fibrosis and cancer, excessive and sustained mechanical stress can promote tumorigenesis and therapeutic resistance. Among these cues, interstitial pressure is particularly significant. In most normal tissues, interstitial fluid pressure (IFP) is slightly negative (approximately -0.3 mmHg), facilitating perfusion. In contrast, solid tumors often exhibit significantly elevated IFP, with values ranging from 20 to 40 mmHg in invasive breast cancer [23]. This buildup of positive pressure impairs blood flow, induces hypoxia, and exerts direct mechanical stress on resident cells. We proposed a model in which mechanical stress deforms the cytoskeleton and activates intracellular signaling cascades, such as the phosphoinositide 3-kinase (PI3K)-Akt pathway, through mechanisms such as integrin clustering and focal adhesion kinase (FAK) signaling, even without direct growth factor receptor activation [24]. PI3K activation is a well-established oncogenic event that promotes GLUT1 expression and glycolytic flux in cancer cells. Thus, mechanically induced PI3K signaling may serve as a direct link between biophysical cues (e.g., high IFP or ECM stiffness) and the early metabolic adaptations characteristic of the Warburg effect. This mechano-metabolic coupling is further amplified by hypoxic conditions, which often accompany high IFP, leading to HIF-1α stabilization. Furthermore, both PI3K-Akt signaling and mechanical stress can upregulate HIF-1α activity through oxygen-independent mechanisms, potentially creating a synergistic feedback loop that locks the glycolytic phenotype [22]. It is important to note that this proposed mechanism, in which mechanical forces directly instigate metabolic reprogramming via pathways such as PI3K, remains a hypothesis that requires rigorous validation. The precise molecular steps linking specific mechanical inputs to changes in metabolite transporter expression and membrane composition are critical areas for future research. Elucidating the interplay between biophysics and metabolism could provide novel therapeutic avenues for targeting the Warburg effect at its inception, potentially before full oncogenic transformation is established in cancer cells.

Oxidative Phosphorylation Revival: A Metabolic Hallmark of Aggressive Tumors

The Warburg effect, which is characterized by a preference for aerobic glycolysis in the presence of oxygen, has traditionally been attributed to defective mitochondrial function. However, current evidence suggests that OXPHOS is not irreversibly impaired but rather suppressed by the metabolic kinetics and regulatory constraints of the cell. This suppression could be reversed by removing these constraints. Rapidly proliferating tissues, including many cancers, rely more heavily on glycolysis for ATP production, not because it is more efficient per molecule of glucose but because it generates ATP at a faster rate per unit time. Additionally, glycolysis supports biosynthesis by providing the intermediates necessary for the production of nucleotides, amino acids, and lipids, which are essential for cell proliferation. Thus, the shift toward glycolysis reflects both the energetic and anabolic demands of rapidly dividing cells in the tumor microenvironment [4,10].

In certain aggressive cancers, such as advanced-stage breast cancer, both OXPHOS and glycolysis contribute to ATP production [25]. We propose that, when the energy demands of tumor cells exceed the maximal ATP output achievable through glycolysis alone, a partial metabolic shift toward OXPHOS is initiated in tumor cells. In tumors with exceptionally high proliferative rates, the ATP-generating capacity of glycolysis may be insufficient, resulting in a decline in intracellular ATP levels. This reduction in cellular energy fell below the threshold required to sustain the PDK activity. Consequently, PDK is inactivated, allowing the PDP to dephosphorylate and reactivate PDH. Reactivated PDH facilitates a greater mitochondrial pyruvate influx, thereby restoring OXPHOS to supplement glycolytic ATP synthesis. Tumors capable of simultaneously engaging in glycolysis and OXPHOS may exhibit particularly high proliferative and invasive potential, as the revival of OXPHOS reflects the extreme energy demands required for rapid growth, driven by successive epigenetic changes.

Notably, not all cancer cells switch to OXPHOS even when they exhibit high rates of proliferation. This metabolic divergence may be attributed to differences in the cell membrane structure, morphology, and specific energy requirements associated with the mode of cell division. Variations in membrane composition can influence nutrient uptake, transporter expression, and mitochondrial function, thereby modulating the balance between glycolytic and oxidative metabolisms. Additionally, the energy cost of proliferation can vary depending on cell type, size, and prioritized biosynthetic pathways. These factors contribute to the heterogeneity observed in cancer cell metabolism and underscore the importance of context-specific regulatory mechanisms. This simultaneous activation of both oxidative OXPHOS and aerobic glycolysis reflects a state of metabolic plasticity that enhances the total ATP output to meet increased energy demands. The re-engagement of mitochondrial respiration during tumor progression is associated with improved biosynthetic capacity, redox homeostasis, and increased metastasis [25].

Importantly, this metabolic flexibility may reduce the effectiveness of therapies targeting glycolysis alone, thereby highlighting the therapeutic challenges posed by metabolically adaptive tumors.

Metabolic Overflow as a Driver of Anabolic Growth in Cancer Cells

A hallmark of cancer cell metabolism is persistently high glycolytic flux coupled with the constitutive activation of anabolic signaling pathways, which together drive biomass accumulation and rapid proliferation. Under normoxic and homeostatic conditions, cells match metabolic flux to biosynthetic demand by storing, degrading, and rerouting excess metabolites. In contrast, proliferating cancer cells sustain elevated glucose uptake and glycolytic activity through the combined action of HIF-1α and PI3K-Akt-mTOR signaling pathways. PI3K activation enhances the Akt-mediated stimulation of mTOR, promotes protein and lipid biosynthesis, and increases ATP production via glucose metabolism [26]. These adaptations supply both the building blocks and the energy needed for growth, although the precise mechanistic link between cell mass accumulation and commitment to division remains under investigation.

Persistent activation of anabolic pathways can cause glycolytic intermediates to accumulate beyond the cell storage and catabolic capacity, resulting in a state of metabolic overflow. In this context, proliferation itself may function as a compensatory mechanism that redistributes excess biosynthetic precursors and relieves intracellular anabolic imbalance, a concept consistent with growth optimization models, although further validation in mammalian systems is required.

Epigenetic Entrenchment in the Warburg Effect

Cancer cells that chronically rely on aerobic glycolysis for intrinsic metabolic reprogramming may undergo epigenetic reinforcement, leading to the stable transmission of the Warburg phenotype to their progeny. This phenomenon, termed epigenetic entrenchment, involves persistent modifications such as DNA methylation, histone acetylation, and lipid membrane remodeling, which collectively reshape chromatin architecture and the gene expression landscape [27]. These epigenetic changes lock the glycolytic state into cell identity, rendering it resistant to metabolic reversion, even in the absence of initiating oncogenic signals.

This stable glycolytic phenotype sustains elevated glucose uptake and lactate production, thereby supporting the substantial biosynthetic and energy requirements for rapid cell proliferation. As proliferation continues, successive generations of daughter cells may acquire additional epigenetic modifications that further enhance the glycolytic flux and anabolic capacity. The persistent accumulation of biosynthetic intermediates such as amino acids, nucleotides, and lipids creates an anabolic burden that drives cells to divide more rapidly as a compensatory mechanism to prevent hypertrophy and preserve metabolic homeostasis. Over time, this self-reinforcing cycle of heightened biosynthesis and accelerated cell division can contribute to unchecked proliferation and progression toward cancer progression.

## Conclusions

The Warburg effect in cancer cells is maintained through biochemical adaptations that circumvent the normal metabolic feedback control mechanisms. Increased glucose uptake results in elevated ATP production and the generation of anabolic intermediates, thereby promoting cellular growth and division. In the absence of adequate feedback inhibition, glycolytic flux and ATP production remain consistently elevated. The overexpression of GLUT1 ensures a continuous supply of glucose, whereas MCT1 and MCT4 upregulation facilitate the rapid export of lactate, thereby preventing product inhibition and sustaining glycolysis. In contrast to normal cells, where lactate accumulation inhibits glycolysis, cancer cells maintain HK and PFK1 activity by enhancing lactate removal. Partial inhibition of glycolysis at downstream bottlenecks leads to the transient accumulation of intermediates, which are redirected into anabolic pathways through mass action to support proliferation.

This metabolic organization can be likened to that of a series of check dams along a rapidly flowing river. Most of the water continues downstream to generate power, whereas a portion is temporarily retained and spontaneously diverted to irrigate nearby fields. Similarly, most glucose-derived carbons in cancer cells are directed toward lactate production for rapid ATP generation, whereas smaller but critical intermediate pools accumulate at bottlenecks and are redirected into biosynthesis. Dynamically regulated metabolic checkpoints ensure that excessive glucose uptake and continuous lactate export provide sustained energy and a steady supply of anabolic precursors. Collectively, these processes fuel the relentless proliferation of cancer cells.
